# High-risk human papillomavirus testing for cervical cancer screening in Uganda: Considering potential harms and benefits in a low-resource setting

**DOI:** 10.1371/journal.pone.0312295

**Published:** 2024-10-23

**Authors:** Marat Sultanov, Jaap A. R. Koot, Geertruida H. de Bock, Marcel J. W. Greuter, Jogchum J. Beltman, Marlieke de Fouw, Janine de Zeeuw, Johnblack Kabukye, Jelle Stekelenburg, Jurjen van der Schans

**Affiliations:** 1 Global Health Unit, Department of Health Sciences, University Medical Center Groningen, University of Groningen, Groningen, Netherlands; 2 Department of Epidemiology, University Medical Center Groningen, University of Groningen, Groningen, Netherlands; 3 Department of Radiology, University Medical Center Groningen, University of Groningen, Groningen, Netherlands; 4 Department of Gynecology, Leiden University Medical Center, Leiden University, Leiden, Netherlands; 5 Uganda Cancer Institute, Kampala, Uganda; 6 Department of Obstetrics and Gynecology, Medical Center Leeuwarden, Leeuwarden, Netherlands; 7 Department of Economics, Econometrics and Finance, Faculty of Economics and Business, University of Groningen, Groningen, Netherlands; Health Directorate, LUXEMBOURG

## Abstract

**Objectives:**

The World Health Organization supports both the screen-and-treat (ST) approach and the screen, triage and treat (STT) approach to cervical cancer screening using high-risk human papillomavirus (hrHPV) testing. For Uganda, the sequence of hrHPV-ST and hrHPV-STT could be similar, with visual inspection with acetic acid (VIA) after positive hrHPV tests in both. To consider potential tradeoffs (overtreatment in ST versus missed cancer cases in STT), we compared hrHPV-STT with VIA triage (STT-VIA), and STT with HPV 16/18 genotyping risk stratification, to hrHPV-ST for Uganda, in terms of overtreatment, cervical cancer incidence, and life years, for the general female population of Uganda.

**Methods:**

A microsimulation model of cervical cancer was adapted. Incremental benefit-harm ratios of STT were calculated as ratios of prevented overtreatment to reduced life years, and to increased cancer cases. Additional scenarios with 20% difference in intra- and inter-screening follow-up between ST and STT were modeled.

**Results:**

Both STT strategies resulted in life year losses on average compared to ST. STT-VIA prevented more overtreatment but led to increased cervical cancer incidence and life year losses. STT-G-VIA resulted in better harm-benefit ratios and additional costs. With better follow-up, STT prevented overtreatment and improved outcomes.

**Discussion:**

For Uganda, the STT approach appears preferrable, if the screening sequences of hrHPV-based ST and STT are similar in practice. While VIA triage alone would reduce overtreatment the most, it could also result in more cancer cases. Risk stratification via genotyping could improve STT. Potential follow-up differences and resource availability should be considered by decision-makers when planning Uganda’s hrHPV-based screening strategy.

## Introduction

In 2021, the World Health Organization (WHO) updated its guidelines for cervical cancer screening to support countries in the global strategy towards elimination of cervical cancer as a public health problem [1, 2]. High-risk human papillomavirus (hrHPV) testing is recommended as the primary screening test with two main follow-up approaches. In the screen-and-treat (ST) approach, all women with a positive hrHPV test receive treatment, while in the screen, triage and treat (STT) approach women receive treatment only after a positive triage test. Both approaches are supported by the WHO, but the country-level implementation of either approach is left to countries’ decision-makers. Instead of identifying precancerous lesions, hrHPV testing identifies hrHPV infections, most of which are transient and do not progress to pre-cancer [3, 4]. Therefore, when considering the choice between hrHPV-based ST and hrHPV-based STT, ST might lead to unnecessary treatment of women without precancerous lesions, with potential rare adverse effects such as increased risk of pre-term birth from excisional treatment [5, 6]. From the policy perspective, overtreatment represents wasted resources, which necessitates consideration of triage to minimize it. However, if loss to follow-up is a significant concern in this comparison, the ST approach may still be preferred, assuming the acceptance of a certain level of overtreatment by the countries’ decision-makers.

On the other hand, STT requires a highly sensitive and specific triage test to minimize numbers of missed pre-cancer cases compared to ST. While high-income countries with established screening programs and infrastructure can afford to transition to cytology and/or colposcopy triaging, low- and middle-income countries (LMICs) often rely on visual inspection of the cervix with acetic acid (VIA) for triage. In these settings, following a positive hrHPV test, VIA is used to determine a woman’s eligibility for ablative treatment in the ST approach, while STT uses VIA as the triage test. This means that in such decision-making context, VIA would be placed in the same position within the screening flow in both ST and STT. However, VIA is subject to high variation in diagnostic accuracy and is dependent on the levels of skill of healthcare workers [7–9]. To make STT more effective, the WHO supports HPV genotyping (16 and 18 genotypes) for risk stratification when it is possible as part of the hrHPV testing system [1], as is the case for commonly used HPV-DNA tests. HPV genotypes 16 and 18 are associated with increased risk of developing cervical cancer compared to other genotypes [10], and such risk stratification has been linked to improvement of screening outcomes in a variety of settings [11–16].

When formulating a national screening program, countries’ decision-makers need to consider these factors and review the potential benefits and harms in the light of the country’s ability to adapt the health system to organize screening according to the WHO recommendations. However, potential screening-related harms, such as overdiagnosis and overtreatment, are rarely quantified by clinical trials due to various reasons [17], while evaluations intended to inform decisions regarding the choice of screening strategy focus on quantifying potential health outcome improvements and cost savings. In this case, decision-analytic modeling can compare the benefit-harm trade-offs of these hypothetical strategies, taking into account the country context. In this study, we chose to focus on Uganda as an example of a resource-constrained setting with an existing VIA-based opportunistic screening policy, where introduction of hrHPV-based strategies utilizing VIA makes practical sense, and where potential harms associated with ST have not been assessed previously. Uganda is a low-income country with a high burden of cervical cancer, accounting for approximately 20% of its cancer incidence and mortality [18], and high hrHPV prevalence estimates ranging from 10% to 40% in women of reproductive age [19, 20]. In practice, its opportunistic VIA-based screening policy has low coverage, resulting in a considerable proportion of underscreened women [21–23]. Previous evaluations found hrHPV testing in a ST approach to be a cost-effective option for Uganda, compared to the existing VIA-based screening with low coverage [24–27]. In our study, we instead aimed to inform the potential choice between hrHPV-based ST and STT for this setting by comparing STT with VIA triage, and STT with HPV 16/18 genotyping and VIA, to the ST approach in terms of life years, costs and overtreatment, for the general female population of Uganda. We did not evaluate the strategies for women living with human immunodeficiency virus (WLHIV), for which the WHO has recommended separate guidelines.

## Methods

This study is conducted in the context of the PREvention and SCReening Innovation Project Toward Elimination of Cervical Cancer (PRESCRIP-TEC) study, which investigates the feasibility of the WHO recommended cervical cancer screening strategy using hrHPV self-sampling in several countries, including Uganda, with a focus on underscreened populations [28].

### Disease model

**Fig 1:**
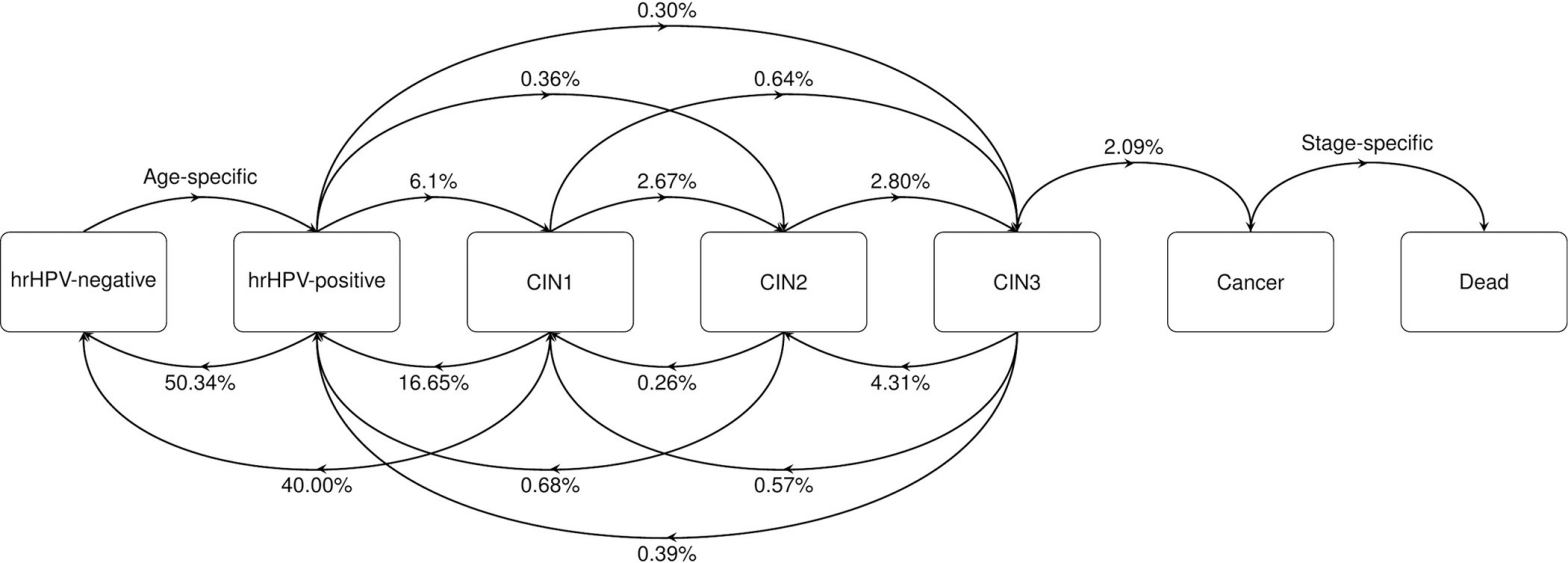
Disease model structure. *The annual transition probabilities are represented in the arrows between disease model states.

This study used a state-transition microsimulation model of cervical cancer progression, which was previously adapted as part of an early model-based evaluation of hrHPV ST strategy for Uganda [29]. The disease model includes 7 mutually exclusive states (“hrHPV-negative”, “hrHPV-positive”, “cervical intraepithelial neoplasia 1 (CIN1)”, “CIN2”, “CIN3”, “Cancer” and “Dead”). The “hrHPV-positive” state refers to hrHPV infection with pre-cancer, while the subsequent states are also hrHPV-infected. Transitions between the states are independently sourced from relevant literature, and validated for existing hrHPV prevalence and cervical cancer incidence in Uganda. The women in the cohort individually progress from age 0 in 1-year steps over a lifetime horizon, transition between states, and enter the screening flows at eligible ages. For the present study, the model was developed to include the distinction between HPV 16/18 genotypes and other hrHPV types for the genotyping strategy by increasing probabilities of progression to pre-cancer states for women with HPV 16/18. The disease model structure with underlying annual transition probabilities is presented in Fig 1, and described in detail in the S1 Appendix.

### Screening strategies

**Fig 2:**
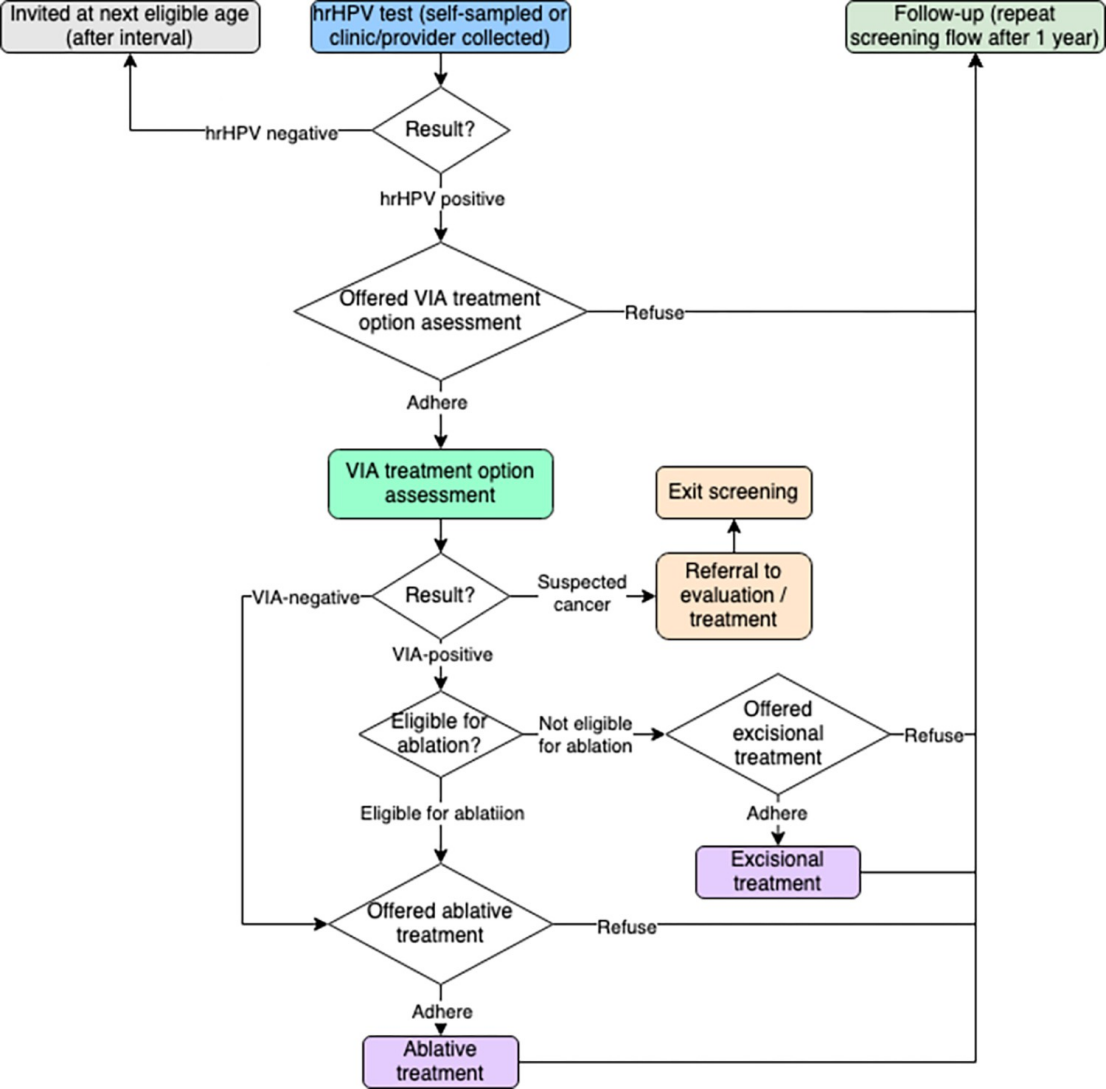
Screening flow—ST strategy.

**Fig 3:**
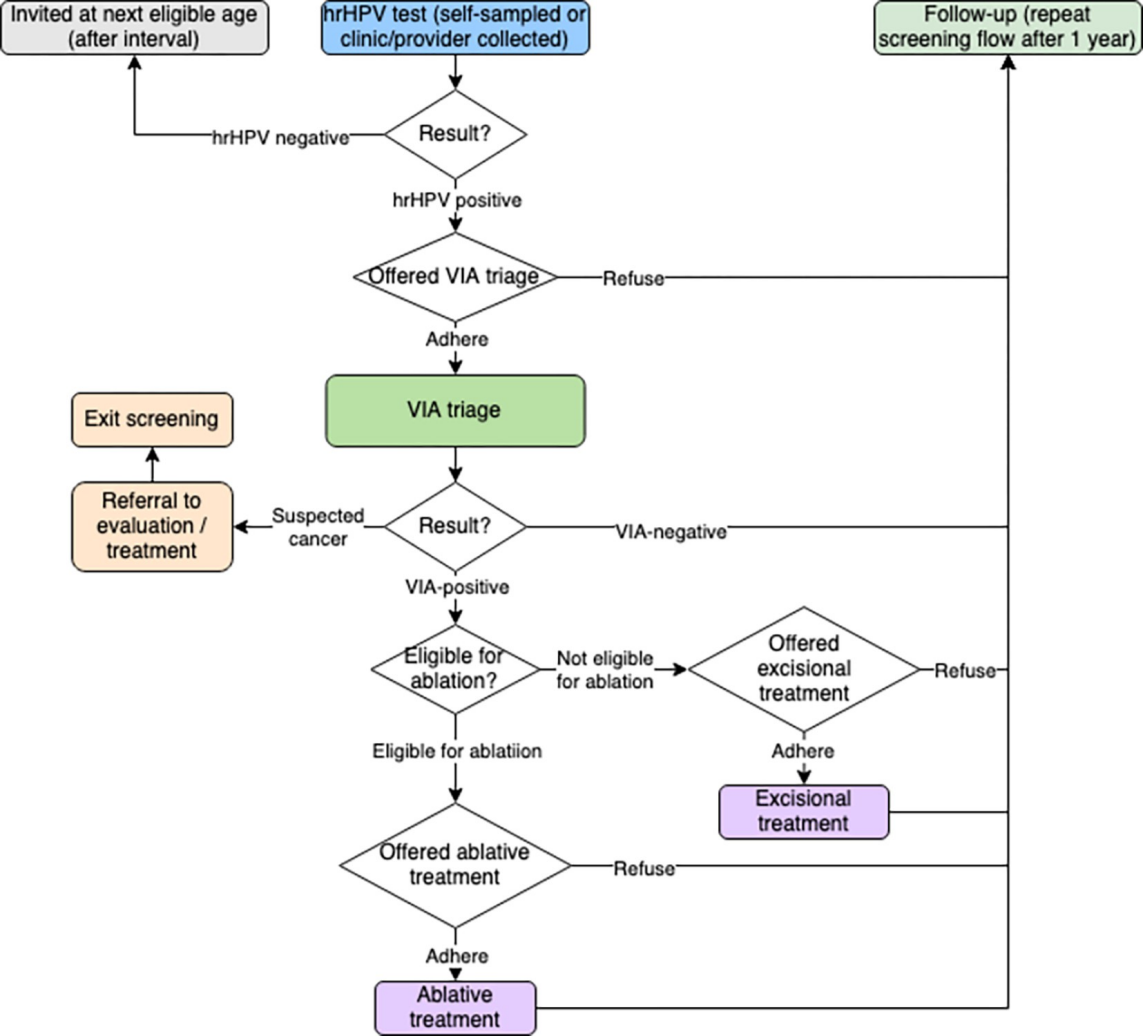
Screening flow—STT-VIA strategy.

**Fig 4:**
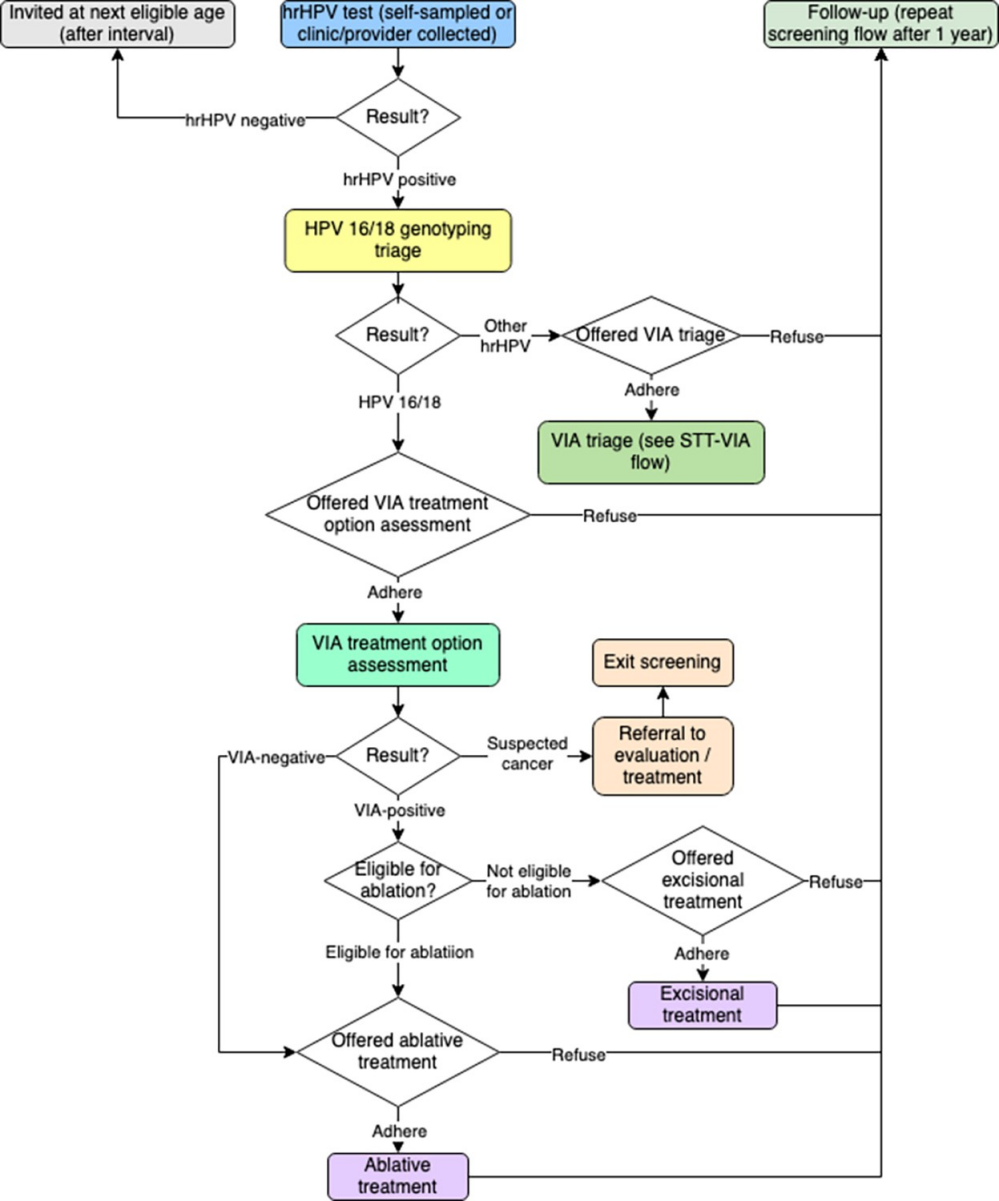
Screening flow—STT-G-VIA strategy.

Since all three strategies are hrHPV-based, we compared two STT strategies to the ST strategy under equivalent screening coverage, focusing on the differences produced by triaging. The three screening strategies modeled in our study are summarized in Table 1. The hrHPV tests in all strategies include a self-sampling option. The corresponding screening flows are presented in Figs 2–4. The genotyping strategy includes VIA triage for women with HPV genotypes that are not HPV 16/18 (“other hrHPV” genotypes). The cost parameters were based on preliminary data from a costing study conducted in several facilities in Uganda as part of PRESCRIP-TEC in 2022. All costs were converted to 2022 international dollars (I$). The evaluation was conducted from the payer (healthcare system) perspective, but did not include programmatic costs of screening, such as cost of interventions to achieve uptake of screening and adherence to follow-up. In the present comparison between three approaches to hrHPV-based screening assuming the same uptake and adherence levels, such costs should, in principle, be broadly similar, whereas they would be more relevant in an evaluation of the hrHPV-based strategies to existing VIA-based screening or absence of screening. The full list of parameters is presented in Table 2.

**Table 1:** Screening strategies.

		**Strategy 1:** hrHPV screen-and-treat (ST)	**Strategy 2:** hrHPV screen, triage and treat—VIA triage (STT-VIA)	**Strategy 3:** hrHPV screen, triage and treat–HPV16/18 genotyping with VIA triage for other hrHPV genotypes (STT-G-VIA)
Population		• A hypothetical cohort of 100 000 women • Individually simulated from age 0 to death • Subject to the same all-cause mortality • Eligible ages^a^ (years): 30–49		
Intervention	Primary test	hrHPV (self-sampled or provider-collected)		
	Risk stratification	-	-	HPV 16/18 genotyping
	Triage test	-	VIA	For HPV 16/18—none
				For other hrHPV genotypes—VIA
	Treatment options	If determined eligible—ablative treatment (thermal ablation / cryotherapy), otherwise—excisional treatment (loop electrosurgical excision procedure)		
	Treatment option determined by	VIA	Determined as part of VIA triage	For HPV 16/18—VIA
				For other hrHPV genotypes—determined as part of VIA triage
	Screening interval (years)	5		
	Follow-up repeat screening (in case of positive hrHPV test result) (years)	1		

^a^The eligible ages are set in accordance with the WHO recommendations (starting at age 30) and the existing Uganda policy extending eligibility to age 49. Due to model logic and 5-year intervals, the last screening round was modeled at age 50.

**Table 2:** Model parameters.

Parameter	Value (base case)	If applicable—sensitivity analysis parameters	Source
**Disease model parameters**			
Age-specific transition probabilities from “hrHPV-negative” to “hrHPV-positive” state	Calibrated to reported prevalence rates	-	[30]
Transition probabilities between “hrHPV-positive”, “CIN1”, “CIN2”, “CIN3”, “Cancer” states	Calibrated to reported cervical cancer incidence rate	-	[18]
HPV 16/18 proportion among hrHPV cases	22.8%	-	[31]
Increased risk modifier for transitions to CIN1/2/3 for HPV16/18	3.6	-	[12]
Cervical cancer stage distribution	Stage I: 11.405% Stage II: 19.505% Stage III: 45.005% Stage IV: 20.805%	-	[32]
Cervical cancer survival	Weibull model fit to reconstructed individual patient dataset)	-	[33]
All-cause age-specific mortality	Global Health Observatory	-	[34]
**Screening-related parameters**			
hrHPV test sensitivity (self-sampled)	92.9%	SD 2.83% (95% CI 87.3%-98.4%)	[35]^a^
hrHPV test specificity (self-sampled)	93.9%	SD 0.28% (95% CI 93.4–94.5%)	
hrHPV test sensitivity (provider-collected)	96.4%	SD 1.79% (95% CI 92.9–99.9%)	
hrHPV test specificity (provider-collected)	94.2%	SD 0.31% (95% CI 93.6–94.8%)	
Proportion of women choosing self-sampling hrHPV test option	83.2%	SD 2.76% (95% CI 77.8–88.6%)	[36]
Proportion of women choosing provider-collected hrHPV test option	26.8%	Complement of proportion of women choosing self-sampling hrHPV test option	
VIA sensitivity	82.4%	SD 2.81% (95% CI 76.3–87.3%)	[9]
VIA specificity	87.4%	SD 4.16% (95% CI 77.1–93.4%)	
Adherence to VIA (as triage test in STT-VIA, as treatment eligibility assessment in ST, as additional triage test in STT-G-VIA)	45.2%	SD 5.8%	[37]
Adherence to ablative treatment	84%	-	[38]
Adherence to excisional treatment			
HPV16/18 genotyping triage sensitivity	49.3%	Assumed 5%	[12]
HPV16/18 genotyping triage specificity	97.5%	Assumed 5%	
Ablative treatment efficacy	74.6%	-	[39]
Excisional treatment efficacy	83.6%	-	
**Cost parameters (I$)**			
hrHPV test (self-sampled)	51.77	-	Preliminary costing data
hrHPV test (provider-collected)	56.77	-	
Ablative treatment	29.4	-	
Excisional treatment	740.7	-	
VIA	12.13	-	
Cervical cancer treatment cost	Stages I-II: 3822.1 Stages III-IV: 2854.9	-	

^a^Parameter unchanged from original model.

### Test assumptions: hrHPV positivity and CIN2+ endpoint

To represent the logic of screening tests in the model, simplified testing assumptions were introduced. When an individual woman entered the screening flow, the hrHPV test result was determined by sensitivity and specificity parameters, and the woman’s current model state. In literature, sensitivity and specificity of hrHPV testing refer to correct identification of women with CIN2+ lesions as hrHPV-positive, since CIN2+ detection is the outcome of interest for evaluations [40, 41]. To account for this, the “hrHPV-positive” state in the disease model referred to hrHPV infection without CIN lesions, while women in “CIN1/2/3” and “Cancer” states were positive for hrHPV infection as well. Thus, the hrHPV test procedure in our model used sensitivity and specificity parameters associated with the CIN2+ endpoint only for women in the corresponding states (“CIN2”, “CIN3”, “Cancer”). For the “hrHPV-positive” and “CIN1” states, perfect accuracy was assumed due to lack of estimates. While such accuracy is obviously not realistic in practice, using the sensitivity and specificity parameters from a different endpoint (CIN2+) was not considered appropriate. Likewise, the sensitivity and specificity parameters of HPV 16/18 genotyping also referred to the CIN2+ endpoint (correct identification of HPV16/18 status in CIN2+ women). Finally, the genotyping procedure in the model similarly applied the sensitivity and specificity parameters to women in CIN2+ states, and assumed perfect accuracy for women in preceding states. An overview of testing logic in the model is provided in S1 Fig. It should be noted that the test accuracy parameters were not stratified by age, for which more granular data on sensitivity and specificity of each test are needed.

### Overtreatment assumptions

In the context of this study, we defined overtreatment as treatment performed on women not in CIN2+ (i.e. women in states “hrHPV-negative”, “hrHPV-positive” or “CIN1”). Thus, overtreatment in the model is a consequence of positive test results, including false positive results of VIA triage in STT, and both true and false positive results of hrHPV testing in ST (where women in states “hrHPV-positive” and “CIN1” are true positive for hrHPV infection, but not the CIN2+ target for treatment).

### Base case analysis

The model was simulated for 100 000 women 100 times for each strategy (due to the probabilistic nature of the microsimulation method). Life years, costs, cervical cancer and overtreatment results were recorded for each simulation, and average results were calculated (means and standard deviations (SD)). Cervical cancer cases were cases of women progressing to state “Cancer” in the state-transition model, and occurred independently of the screening flows (which they left if cancer was detected during screening). Incremental results for the comparisons (STT-VIA versus ST and STT-G-VIA versus ST) were calculated using the differences in mean life years and difference in mean costs per woman. For overtreatment, both absolute and relative reductions in overtreatment compared to ST were calculated. We calculated incremental benefit-harm ratios as ratios of prevented overtreatment cases to lost life years and to additional cervical cancer cases. Discounting for costs was applied at 3% annual rate starting from age 30 (the first age eligible for screening), which is commonly applied in health economic evaluations.

### Sensitivity analysis

One-way probabilistic sensitivity analysis was performed for selected parameters, including sensitivity and specificity of hrHPV test, VIA and HPV 16/18 genotyping, and adherence to VIA (follow-up). Details of sensitivity analysis parameters are provided in the Table 2.

### Follow-up scenario analysis

We differentiated between two types of loss to follow-up in our model. Inter-screening loss to follow-up was represented by coverage of 1-year repeat screening for women who had received a positive hrHPV test result in the previous year (regardless of subsequent steps in the screening flow). A recent analysis stressed the importance of high 1-year follow-up rates for hrHPV-based STT strategies to be effective compared to ST [42]. On the other hand, intra-screening loss to follow-up was determined by adherence to VIA assessment (in ST) or VIA triage (STT-VIA and STT-G-VIA), which represented the proportion of women dropping out of the screening flow after a positive hrHPV test result (Figs 2–4). It should be noted that even with the same parameter of VIA adherence, the absolute numbers of intra-screening loss to follow-up cases in STT would be lower compared to ST due to fewer women having the option to refuse treatment in the screening flow and drop out. However, since the purpose of invitation for VIA in the ST strategy would be communicated to the woman as a treatment visit, as opposed to a test, VIA adherence could also differ in practice between ST and STT even with a seemingly similar sequence of steps. Negative VIA triage results and subsequent lack of treatment have been reported as potential source of confusion for women [43]. Acceptability of triage and associated factors will need to be studied more in the future among underscreened populations [44].

Taking these factors into account, we ran additional simulations for hypothetical “worst case” and “best case” scenarios to examine the impact of intra- and inter-screening follow-up. “Worst case” was defined as intra-screening follow-up of 25.2% and inter-screening follow-up of 80%, representing 20% absolute reduction from base case parameters (Table 2), while “best case” was defined as intra-screening follow-up of 45.2% and inter-screening follow-up of 100%. We note that these scenarios were not meant to represent realistic follow-up rates, but rather the difference between hypothetical ST and STT scenarios, since the base case analysis compared them under equivalent (100%) rates.

## Results

### Base case analysis

The simulation results are presented in Table 3. Both STT-VIA and STT-G-VIA resulted in loss of life years on average compared to ST, albeit with high variation around the mean difference. STT-VIA resulted in cost reduction compared to ST, while STT-G-VIA produced additional costs. STT-VIA was slightly more effective in terms of reducing overtreatment than STT-G-VIA at the cost of higher loss of life years and increased cervical cancer incidence. Replacing ST with STT-G-VIA resulted in approximately 40% fewer additional cancer cases as opposed to replacing ST with STT-VIA without genotyping risk stratification. In terms of cervical cancer incidence, the ST strategy resulted in the lowest incidence rate (mean rate 13.78 per 100 000 woman-years at risk), while the corresponding incidence rate of the STT-VIA strategy was 9.8% higher (mean rate 15.13 per 100 000 woman-years at risk) compared to ST. In terms of harms and benefits, STT-G-VIA resulted in better harm-benefit ratios than STT-VIA, with more cases of overtreatment avoided per lost life year and per additional cervical cancer case.

**Table 3:** Base case results.

Outcome	ST	STT-VIA	STT-G-VIA
	Mean (SD)		
Total life years per woman (life expectancy)	69.00 (0.09)	68.97 (0.09)	68.98 (0.09)
Incremental life years per woman	Ref	-0.04 (0.07)	-0.03 (0.09)
Lifetime costs per woman, I$	123.36 (2.23)	120.66 (2.33)	129.87 (2.52)
Lifetime incremental costs per woman, I$	Ref	-2.69 (0.76)	6.52 (0.68)
Total number of overtreatment cases	78 928 (997)	10 590 (378)	23 786 (585)
Incremental overtreatment cases per woman^a^^,^^b^	Ref	-0.68 (0.00)	-0.55 (0.00)
Overtreatment proportion relative to ST^c^	Ref	0.13 (0.00)	0.30 (0.00)
Total number of cases of intra-screening loss to follow-up	140 734 (411)	137 481 (563)	137 327 (449)
Incremental cases of intra-screening loss to follow-up	Ref	-3252 (657)	-3 407 (589)
Total number of cervical cancer cases	951 (254)	1 044 (265)	1 006 (255)
Incremental cervical cancer cases	Ref	92.57 (64.30)	55.01 (40.58)
Cervical cancer incidence rate per 100 000 woman-years at risk	13.78 (0.49)	15.13 (0.69)	14.59 (0.41)
Incremental benefit-harm ratio (number of overtreatments avoided per 1 life year lost, relative to ST)	Ref	18.46	21.68
Incremental benefit-harm ratio (number of overtreatments avoided per 1 additional cervical cancer case, relative to ST)	Ref	738.23	1 002.40

^a^Average absolute number of overtreatments prevented across a woman’s lifetime in the population

^b^Ratio of total number of overtreatment cases in STT to total number of overtreatment cases in ST

### Sensitivity analysis

The results of one-way sensitivity analysis for selected model parameters are presented in the S2–S5 Figs for two outcomes (incremental life years and incremental overtreatment cases). The sensitivity parameter of hrHPV test produced the largest differences in terms of incremental life years. For both STT strategies, the analysis resulted in gains and losses of life years, which was not achieved on average under base case assumptions. As expected, adherence to VIA (affecting intra-screening follow-up) and specificity of VIA were the most influential variables for the overtreatment outcomes, since all three strategies included the VIA step before treatment. However, the range of overtreatment results when varying VIA adherence and specificity remained negative (max -0.46, min -0.86 incremental cases per woman), and the corresponding interpretation (reduction of overtreatment in STT compared to ST) would not change. This contrasts with the life years results, where (along with all other parameters) both positive and negative incremental life years were observed (max 0.25, min -0.30), which illustrated the impact of uncertainty on potential interpretation of differences between the strategies. In case of positive life year results (gained life years in STT compared to ST), the interpretation of benefit-harm ratios of STT would change, since the “harm” part in the base case analysis was represented by loss of life years (negative incremental values) compared to ST.

### Follow-up scenario analysis

The follow-up scenario analysis (Table 4) revealed that with higher inter-screening and intra-screening follow-up STT strategies became preferable to ST, simultaneously resulting in reductions in overtreatment and improvement of health outcomes (gaining life years and preventing cervical cancer cases).

**Table 4:** Follow-up scenario results.

Outcome	STT-VIA vs ST		STT-G-VIA vs ST	
	Best case ST, worst case STT	Worst case ST, best case STT	Best case ST, worst case STT	Worst case ST, best case STT
	Mean (SD)			
Incremental life years per woman^a^	-0.10 (0.09)	0.04 (0.11)	-0.05 (0.10)	0.04 (0.10)
Incremental overtreatment cases per woman^b^	-0.73 (0.00)	-0.33 (0.00)	-0.59 (0.00)	-0.27 (0.00)
Incremental cervical cancer cases^c^	338 (49)	-139 (44)	189 (45)	-104 (54)
Incremental benefit-harm ratio (per 1 life year lost/gained)^d^	7.10	-7.76	12.66	-5.94
Incremental benefit-harm ratio (per 1 additional/prevented cervical cancer case)^e^	216.03	-240.43	313.84	-255.00

^a^Negative values represent lost life years compared to ST

^b^Negative values represent averted overtreatment cases compared to ST

^c^Negative values represent prevented cervical cancer cases compared to ST

^d^Negative ratio is interpreted as overtreatment cases prevented per 1 life year gained

^e^Negative ratio is interpreted as overtreatment cases prevented per 1 cervical cancer case prevented

## Discussion

### ST versus STT: Balancing tradeoffs

In the present study, we modeled hypothetical hrHPV-based ST and STT strategies under similar implementation conditions for Uganda. The ST strategy predictably resulted in lower cervical cancer incidence compared to STT through increased treatment and would be the preferred strategy without considering overtreatment. Both triage strategies appeared to reduce overtreatment substantially when compared to ST. However, this was achieved at the cost of losing life years due to missed pre-cancer cases, which resulted in additional cervical cancer cases and associated mortality. HPV 16/18 genotyping with VIA triage produced smaller reductions in overtreatment than VIA triage alone (0.55 vs 0.68), but also resulted in fewer additional total cervical cancer cases (55.01 vs 92.57). Moreover, it also incurred additional costs, while the VIA triage alone resulted in cost savings compared to ST.

Whether the associated increase in cervical cancer incidence and loss of life years in STT are acceptable will depend on the decision-makers’ priorities. Currently, there are no commonly accepted threshold or reference levels for overtreatment rates for cervical cancer screening programs [45], unlike, for example, cost-effectiveness / willingness-to-pay thresholds. While reduction of cervical cancer burden is obviously the key priority of screening programs, the potential negative consequences associated with treatment have to be taken into account. However, while short-term side effects and direct complications of treatment may be rare, data on long-term adverse events related to fertility and obstetric outcomes is lacking. The Cochrane meta-analysis [46] highlights the low quality of available evidence on these risks of treatment. We therefore chose not to model adverse events and instead presented benefit-harm ratios, focusing on overtreatment itself as the negative outcome that represents wasted resources, which are already limited in settings like Uganda, and potential harm. For screening strategies evaluated in this study, the ST approach could result in significant additional pressure on the healthcare system, considering high acceptability of treatment among women in LMICs [47], and Uganda in particular [48].

As stated previously, if the follow-up rates are lower for STT than ST with the same sequence of tests due to women’s preference for treatment over another test, the ST approach could still be preferred in this comparison. However, considering the higher hrHPV prevalence among younger women and pregnancy-related risks associated with excisional treatment in particular [6, 49], targeted triaging for these women could potentially be a better option to balance the tradeoffs. More research is needed on long-term screening- and treatment-related adverse events to inform targeted recommendations.

### Importance of VIA in this comparison

In our sensitivity analysis, variation in VIA-related parameters affected overtreatment outcomes more than other parameters, although the model appeared relatively robust for this outcome in general. Since VIA accuracy depends on the skill level of healthcare workers, achieving high specificity will be necessary for a substantial reduction in overtreatment. However, this applies to both STT and ST, unless quality of VIA differs significantly between the two approaches, and thus should not affect the choice between STT and ST in this setting. It should also be noted that the relatively high sensitivity and specificity parameter values for CIN2+ detection for VIA (82.4% & 87.4%) in our analysis were based on pooled estimates from a meta-analysis of African studies [9]. In practice, such accuracy may not be realistic or sustainable, particularly during initial implementation of new screening programs. Achieving high quality of VIA will require additional costs and human resources, including training of workers [50] and monitoring of VIA quality, which should be taken into account in future evaluations. Developments in artificial intelligence as decision support systems for VIA could potentially help reduce these costs and help ensure sufficient accuracy [51, 52].

### Follow-up implications

A recent analysis [42] suggested that with high follow-up rates STT strategies may become equivalent to ST in terms of life years, while simultaenously reducing overtreatment. In our “best-worst” case analysis, the ST approach similarly appeared to become preferable to STT if its follow-up rates were higher. On the other hand, the “worst case” scenario with 20% reduction in intra-screening and inter-screening follow-up considerably worsened the outcomes (incremental life years: -0.10 vs -0.04 for STT-VIA, -0.05 vs -0.03 for STT-G-VIA; incremental cancer cases: 338.08 vs 92.57 for STT-VIA, 188.79 vs 55.01 for STT-G-VIA). While high follow-up rates will invariably be necessary for either ST or STT approach to hrHPV-based screening to succeed in this setting, our results suggest that the STT approach could potentially benefit more from investing in efforts to increase follow-up compared to ST, to the point of achieving parity or even improvement in cervical cancer incidence and life years, which would make the choice between ST and STT redundant. Health systems strengthening will be important in this context and could help make STT more favorable. It should also be noted that our comparison assumed the same proportion of women choosing the self-sampling option for hrHPV testing, and did not include programmatic costs of follow-up. Since a self-sampling program involves collection of samples from women’s homes and transport to laboratories, and a system to communicate test results to the women and invite them for a follow-up, the associated costs could vary depending on the context and set-up of the program. Further data on women’s preferences in these settings and costs of achieving adherence to follow-up would help inform detailed country-level assessments.

### Genotyping: Worth the potential additional cost?

HPV 16/18 genotyping has been linked to improved outcomes in a variety of settings when compared to absence of screening or VIA-based screening [11–16]. While the results in terms of benefit-harm ratios were promising in our analysis, the STT-G-VIA strategy also appeared to cost more compared to ST. The reason for this difference was the relatively low sensitivity parameter used (49.5%) for HPV 16/18 genotyping in the model, based on a sub-analysis of trial data [12]. This resulted in significant numbers of women at actual higher risk of progression to cancer (HPV 16/18 types) being incorrectly identified as “other hrHPV” types (false negatives). These women in the model underwent the same screening flow as STT-VIA, being subject to a similarly low rate of overtreatment but having higher underlying risks of cancer progression (compared to risks of progression for women in these states in STT-VIA without risk stratification), which in turn made the treatment less cost-effective on average for this subgroup. At the same time, the women identified as HPV 16/18 (both true and false positives) proceeded through the ST flow, which was more expensive on average compared to STT-VIA.

Furthermore, the economic value of genotyping was also impacted by the proportion of HPV 16/18 infections present in CIN2+ lesions, for which we used an assumption based on proportion of cervical cancers caused by HPV 16/18 (73.5%). Depending on disease progression (relating to how HPV 16/18 accelerates progression to CIN and cancer), the effect of HPV 16/18 genotyping on reducing cancer incidence could vary, since overtreatment of CIN1 cases with HPV 16/18 prevents more cancer cases compared to overtreatment of CIN1 without HPV type risk stratification in STT-VIA. This was also evident in our benefit-harm ratio results, where each additional cervical cancer case in STT-G-VIA caused 36% more overtreatment cases compared to STT-VIA.

In this context, it is important to consider that cervical cancer screening is underfunded in Uganda [53], where out-of-pocket expenditures constitute a large proportion of screening and treatment-related costs [54, 55]. Thus, this potential additional cost of HPV 16/18 genotyping may not be found acceptable by the country’s decision-makers, making VIA triage without genotyping preferable instead. More country-specific data on genotype distribution and associated risks of progression to CIN2+ would help provide a more accurate estimate of the benefit of HPV 16/18 genotyping to inform targeted approaches.

Finally, the definition of overtreatment could affect the interpretation of results. In STT-G-VIA, a quarter of overtreatment cases were treatments performed on women with CIN1, while for the STT-VIA and ST strategies the corresponding proportions were approximately 10% and 12% (with the rest of overtreatments performed either on hrHPV-infected women without CIN lesions or hrHPV-negative women). For some stakeholders, treatments performed on women without any cervical abnormalities could arguably be considered less acceptable or more wasteful, making genotyping more favorable compared to VIA triage alone.

### Strengths and limitations

Our study adds to literature on evaluation of hrHPV-based screening strategies by considering their potential impact in a broader context of benefits and harms, as well as the costs and effects, with a focus on a specific decision context of ST and STT in a country with existing VIA. However, there are several limitations to our study. Our analysis quantifies the health outcomes in terms of life years, without adjusting for health-related quality of life. Depending on the impact of treatment procedures on women’s quality of life, reduction in overtreatment could be evaluated differently in this context. Our analysis also adopted a simplified representation of overtreatment. If risks of cervical cancer progression were modeled differently depending on age, overtreatment of hrHPV infection or CIN1 lesions could be evaluated as more acceptable for age groups with increased risk of progression from those states. Furthermore, we adopted a simplified representation of cancer progression without modeling individual transition between cancer stages, which requires detailed staging data. Therefore, the impact of screening on downstaging of cervical cancer is not directly expressed in the life years results of our model, apart from the reduction of cancer cases from treated pre-cancer cases. However, for an underscreened population, in which most women would be screened for the first time, the implementation of screening would result in revealing real prevalence of cancer, while the downstaging effect would become visible with continued high uptake and repeated screenings. We also limited our analysis to population of women without human immunodeficiency virus, known to increase the risk of hrHPV progression to pre-cancer, which would require a separate decision model. As is the case with WHO recommendations, dedicated analyses need to be conducted for WLHIV to inform targeted screening approaches, which may be particularly relevant for Uganda given the prevalence of HIV in the country.

## Conclusions

Our analysis provides valuable information to stakeholders in Uganda and other LMICs with existing opportunistic VIA-based screening policies, where the successful implementation of hrHPV-based screening will be crucial to achieve global cervical cancer elimination goals. The results could serve the country’s decision-makers in choosing the most suitable approach to hrHPV-based screening for their setting and considering their own priorities in balancing the potential harms and benefits of triaging.

Under the same implementation conditions as ST, the STT approach to hrHPV-based cervical cancer screening in the low-resource context of Uganda could result in substantially lower overtreatment at the cost of loss of life years and missed pre-cancer cases. Decision-makers need to consider the benefits and harms associated with STT and ST prior to implementation. Adding HPV 16/18 genotyping to STT for risk stratification could potentially represent a middle ground between ST and STT with VIA triage alone, albeit with additional costs. Further research into potential differences in follow-up rates between similar hrHPV-based ST and STT approaches utilizing VIA may inform these assumptions in planning the implementation in this setting.

## Supporting information

S1 Checklist CHEERS checklist. (PDF)

S1 Fig Testing logic. (TIF)

S2 Fig Sensitivity analysis results—STT-VIA, incremental life years. (TIF)

S3 Fig Sensitivity analysis results–STT-G-VIA, incremental life years. (TIF)

S4 Fig Sensitivity analysis results–STT-VIA, incremental overtreatment cases. (TIF)

S5 Fig Sensitivity analysis results–STT-G-VIA, incremental overtreatment cases. (TIF)

S1 Appendix (DOCX)
