# Supplementary material for: High-risk human papillomavirus testing for cervical cancer screening in Uganda: Considering potential harms and benefits in a low-resource setting
Source: PLoS One. 2024 Oct 23;19(10):e0312295. doi: 10.1371/journal.pone.0312295 (PMC11498676; doi:10.1371/journal.pone.0312295)
Supplement: S1 Checklist — (PDF) [file pone.0312295.s001.pdf]

CHEERS 2022 Checklist

Topic	No.	Item	Location where item is reported
**Title**			
	1	Identify the study as an economic evaluation and specify the interventions being compared.	Title page
**Abstract**			
	2	Provide a structured summary that highlights context, key methods, results, and alternative analyses.	Abstract
**Introduction**			
**Background and objectives**	3	Give the context for the study, the study question, and its practical relevance for decision making in policy or practice.	"Introduction"
**Methods**			
**Health economic analysis plan**	4	Indicate whether a health economic analysis plan was developed and where available.	Not reported
**Study population**	5	Describe characteristics of the study population (such as age range, demographics, socioeconomic, or clinical characteristics).	"Disease model", Table 1
**Setting and location**	6	Provide relevant contextual information that may influence findings.	Table 1
**Comparators**	7	Describe the interventions or strategies being compared and why chosen.	"Screening strategies", Table 1, Figs 2-4
**Perspective**	8	State the perspective(s) adopted by the study and why chosen.	"Screening strategies"
**Time horizon**	9	State the time horizon for the study and why appropriate.	"Disease model"
**Discount rate**	10	Report the discount rate(s) and reason chosen.	"Base case analysis"
**Selection of outcomes**	11	Describe what outcomes were used as the measure(s) of benefit(s) and harm(s).	"Base case analysis"
**Measurement of outcomes**	12	Describe how outcomes used to capture benefit(s) and harm(s) were measured.	"Base case analysis"

Topic	No.	Item	Location where item is reported
**Valuation of outcomes**	13	Describe the population and methods used to measure and value outcomes.	“Base case analysis", Table 2
**Measurement and valuation of resources and costs**	14	Describe how costs were valued.	‘Screening strategies", Table 2
**Currency, price date, and conversion**	15	Report the dates of the estimated resource quantities and unit costs, plus the currency and year of conversion.	"Screening strategies"
**Rationale and description of model**	16	If modelling is used, describe in detail and why used. Report if the model is publicly available and where it can be accessed.	"Disease model", Figure 1, S1 Appendix
**Analytics and assumptions**	17	Describe any methods for analysing or statistically transforming data, any extrapolation methods, and approaches for validating any model used.	S1 Appendix ("Model calibration")
**Characterising heterogeneity**	18	Describe any methods used for estimating how the results of the study vary for subgroups.	Not reported
**Characterising distributional effects**	19	Describe how impacts are distributed across different individuals or adjustments made to reflect priority populations.	Not reported
**Characterising uncertainty**	20	Describe methods to characterise any sources of uncertainty in the analysis.	“Sensitivity analysis"
**Approach to engagement with patients and others affected by the study**	21	Describe any approaches to engage patients or service recipients, the general public, communities, or stakeholders (such as clinicians or payers) in the design of the study.	Not reported
**Results**			
**Study parameters**	22	Report all analytic inputs (such as values, ranges, references) including uncertainty or distributional assumptions.	Table 2
**Summary of main results**	23	Report the mean values for the main categories of costs and outcomes of interest and summarise them in the most appropriate overall measure.	Table 3
**Effect of uncertainty**	24	Describe how uncertainty about analytic judgments, inputs, or projections affect findings. Report the effect of choice of discount rate and time horizon, if applicable.	“Sensitivity analysis"

Topic	No.	Item	Location where item is reported
**Effect of engagement with patients and others affected by the study**	25	Report on any difference patient/service recipient, general public, community, or stakeholder involvement made to the approach or findings of the study	Not reported
**Discussion**			
**Study findings, limitations, generalisability, and current knowledge**	26	Report key findings, limitations, ethical or equity considerations not captured, and how these could affect patients, policy, or practice.	"Discussion"
**Other relevant information**			
**Source of funding**	27	Describe how the study was funded and any role of the funder in the identification, design, conduct, and reporting of the analysis	Provided through the submission system
**Conflicts of interest**	28	Report authors conflicts of interest according to journal or International Committee of Medical Journal Editors requirements.	Provided through the submission system

From: Husereau D, Drummond M, Augustovski F, et al. Consolidated Health Economic Evaluation Reporting Standards 2022 (CHEERS 2022) Explanation and Elaboration: A Report of the ISPOR CHEERS II Good Practices Task Force. Value Health 2022;25. doi:10.1016/j.jval.2021.10.008
