# Supplementary figures and images for: High-risk human papillomavirus testing for cervical cancer screening in Uganda: Considering potential harms and benefits in a low-resource setting
Source: PLoS One. 2024 Oct 23;19(10):e0312295. doi: 10.1371/journal.pone.0312295 (PMC11498676; doi:10.1371/journal.pone.0312295)

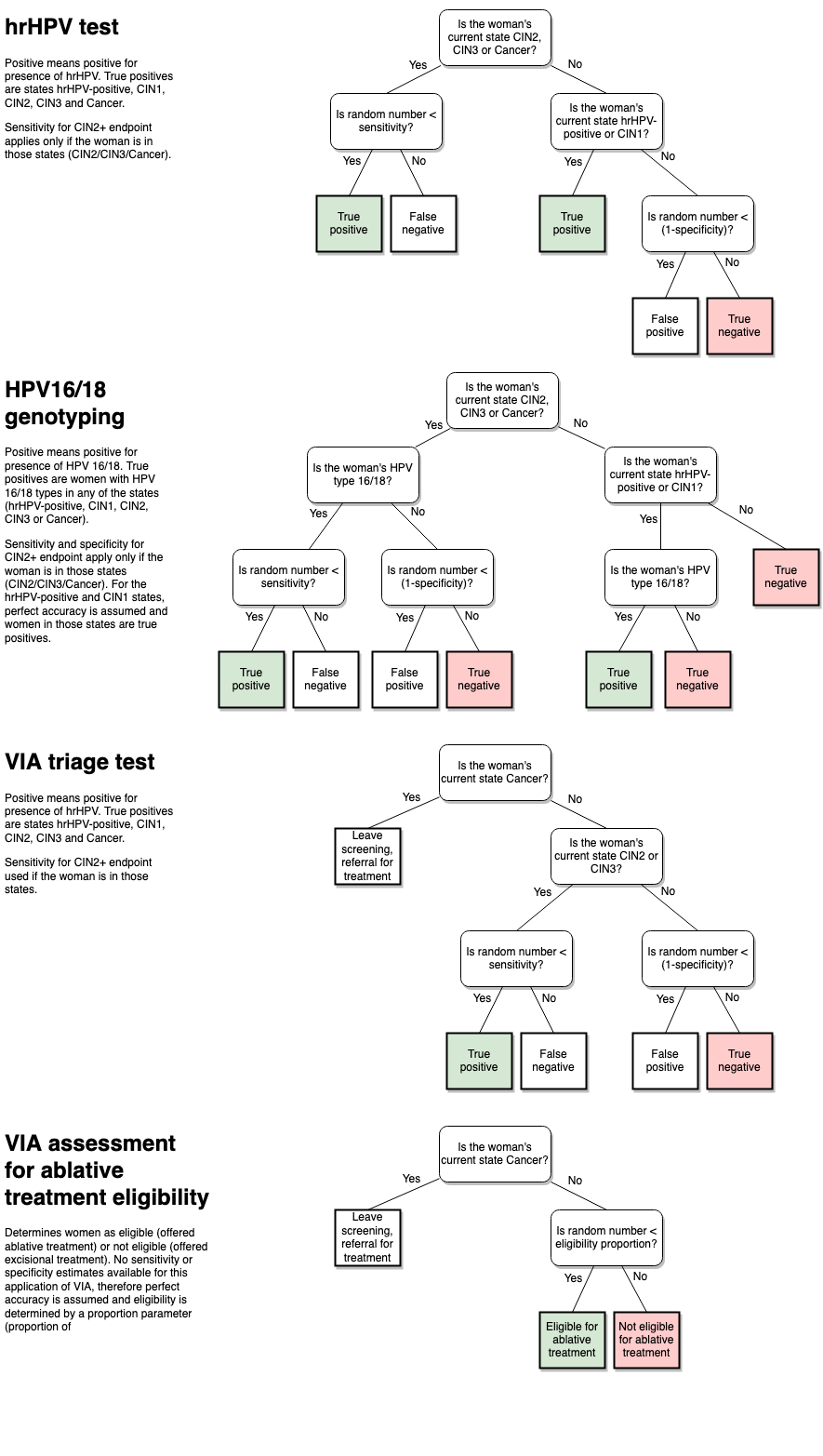

Supplement: S1 Fig — (TIF) [file pone.0312295.s002.tif]

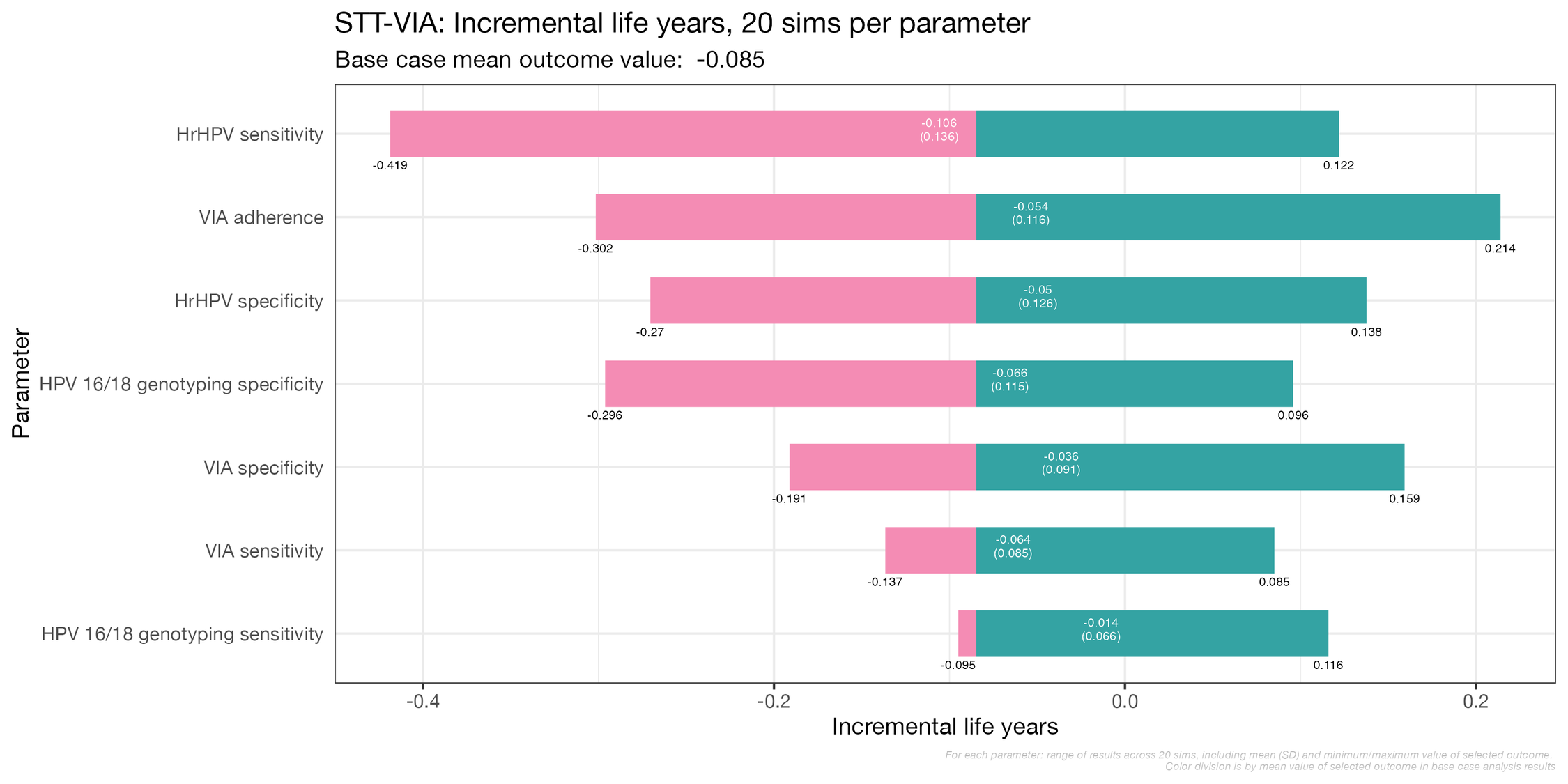

Supplement: S2 Fig — (TIF) [file pone.0312295.s003.tif]

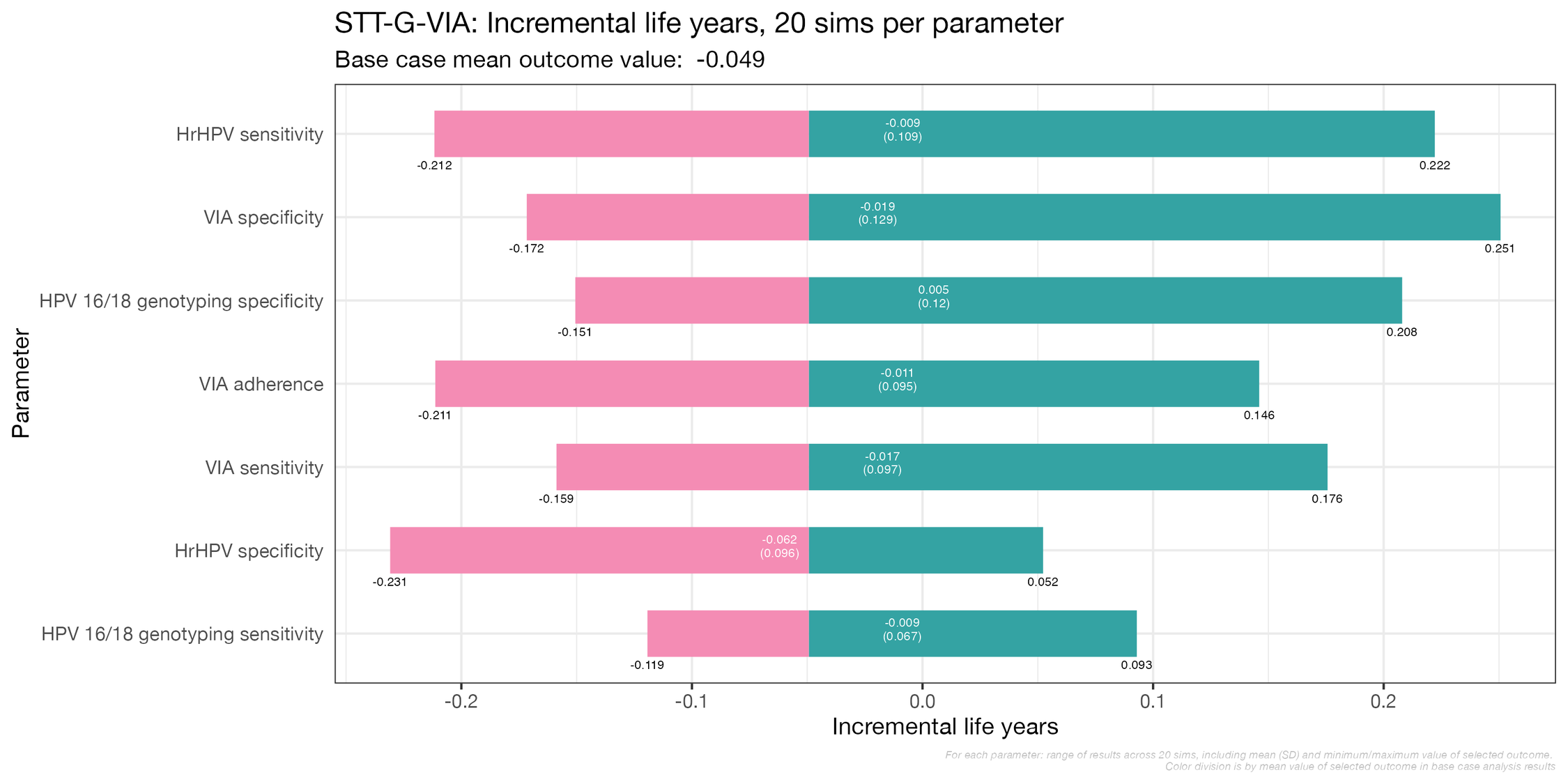

Supplement: S3 Fig — (TIF) [file pone.0312295.s004.tif]

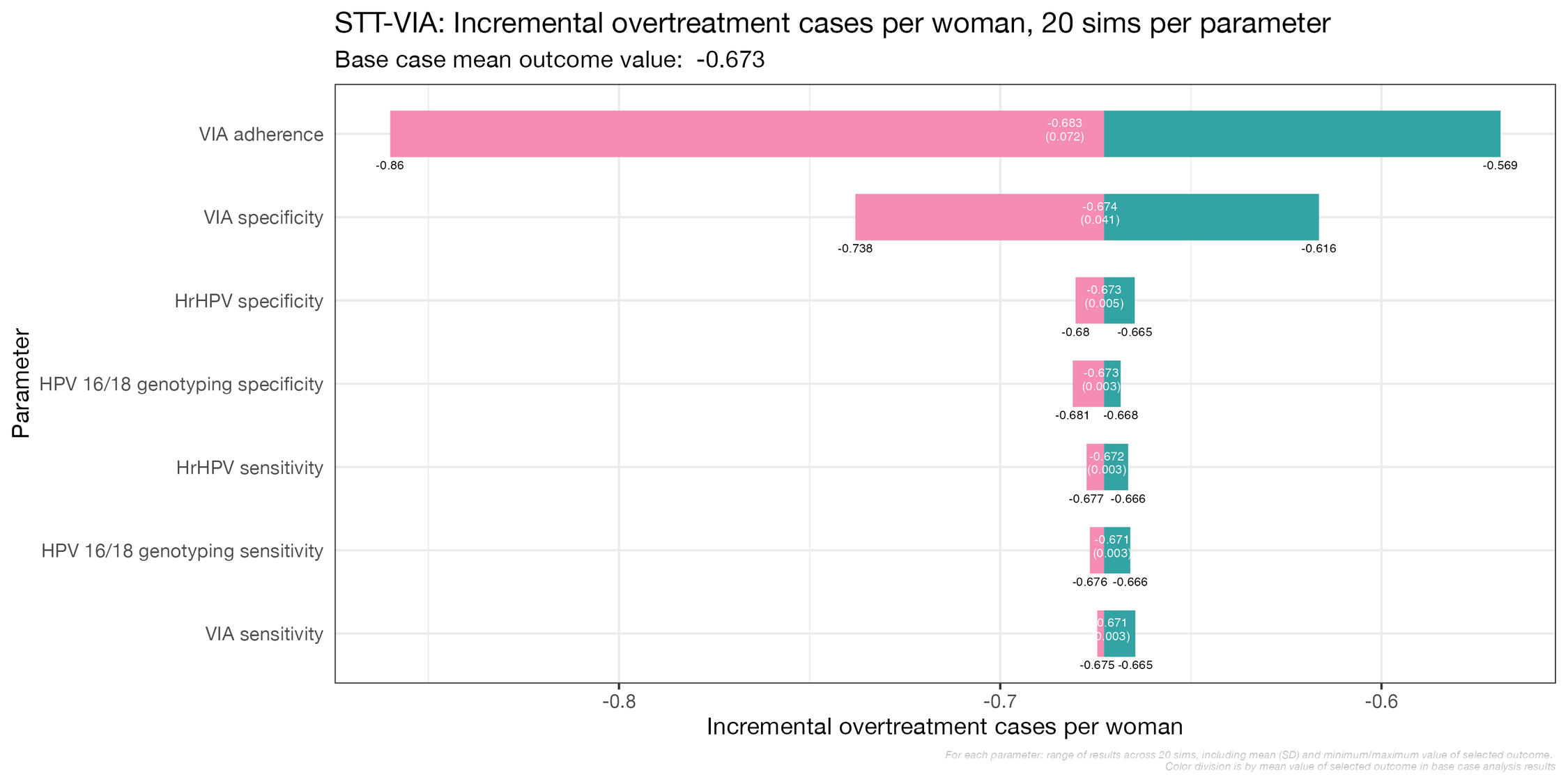

Supplement: S4 Fig — (TIF) [file pone.0312295.s005.tif]

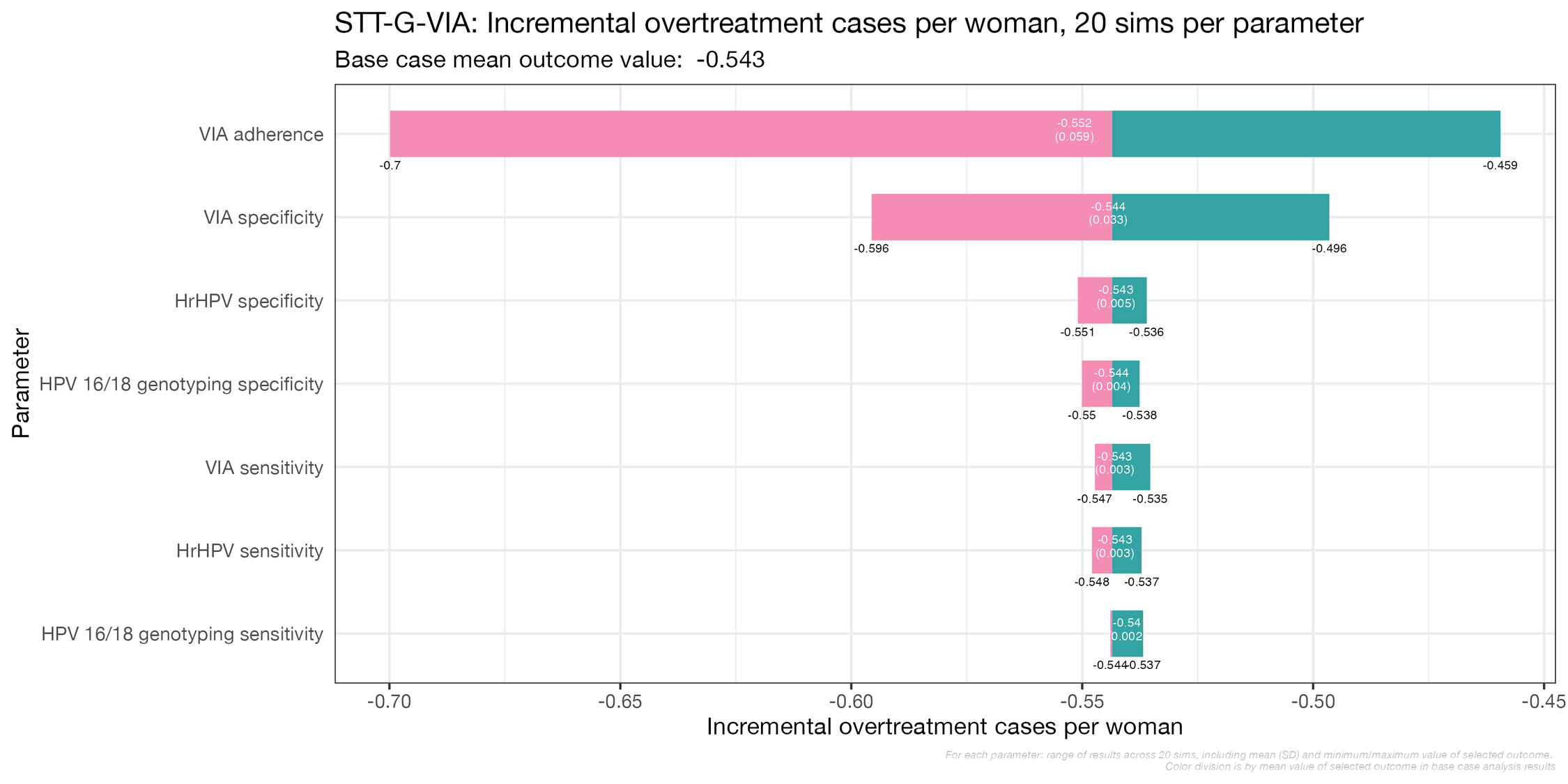

Supplement: S5 Fig — (TIF) [file pone.0312295.s006.tif]
