# Supplementary material for: High-risk human papillomavirus testing for cervical cancer screening in Uganda: Considering potential harms and benefits in a low-resource setting
Source: PLoS One. 2024 Oct 23;19(10):e0312295. doi: 10.1371/journal.pone.0312295 (PMC11498676; doi:10.1371/journal.pone.0312295)
Supplement: S1 Appendix — (DOCX) [file pone.0312295.s007.docx]

**S1 Appendix**

Supporting information for *“High-risk human papillomavirus testing for cervical cancer screening in Uganda: considering potential harms and benefits in a low-resource setting”*

**Authors:** Marat Sultanov^*^, Jaap A.R. Koot, Geertruida H. de Bock, Marcel J.W. Greuter, Jogchum J. Beltman, Marlieke de Fouw, Janine de Zeeuw, Johnblack Kabukye, Jelle Stekelenburg, Jurjen van der Schans

*Corresponding author: m.sultanov@umcg.nl (MS)

**Disease model description**

The disease model included 7 mutually exclusive states:

- High-risk human papillomavirus (hrHPV)-negative;
- HrHPV-positive;
- Cervical intraepithelial neoplasia (CIN) 1;
- CIN 2;
- CIN 3;
- Cancer;
- Dead.

The key assumptions involved in the disease model are described as follows:

- Progression to any cervical intraepithelial neoplasia (CIN) states (CIN1, CIN2 or CIN3), occurs only from the hrHPV-positive state;
- Progression to cervical cancer occurs only from CIN3 state;
- There is no direct regression from CIN2 or CIN3 to hrHPV-negative;
- Cervical cancer is modeled as a single state, with each woman entering the state being assigned a cancer stage (according to International Federation of Gynecology and Obstetrics (FIGO) system), based on reported stage distribution.

The annual transition probabilities from the original disease model drawn from relevant literature are provided in Table S1. These values were used as the initial values for calibration (see “Calibration” section).

**Table S1. Initial transition probabilities**

| ***to from*** | **hrHPV-negative** | **hrHPV-positive** | **CIN1** | **CIN2** | **CIN3** | **Cancer** |
| --- | --- | --- | --- | --- | --- | --- |
| **hrHPV-negative** | - | Age-specific | 0 | 0 | 0 | 0 |
| **hrHPV-positive** | 0.5034 [1] | - | 0.0610 [2] | 0.0034 [3] | 0.0072 [3] | 0 |
| **CIN1** | 0.4000 [4] | 0.1600 [4] | - | 0.0242 [4] | 0.0047 [4] | 0 |
| **CIN2** | 0 | 0.0073 [5] | 0.0244 [5] | - | 0.0474 [5] | 0 |
| **CIN3** | 0 | 0.0025 [6] | 0.0074 [6] | 0.0486 [6] | - | 0.0400 [7] |
| **Cancer** | 0 | 0 | 0 | 0 | 0 | - |

The background mortality probabilities (i.e. transition probabilities to “Dead” state from all states, except for “Cancer” state) were age-specific and determined using the latest available age-specific mortality data from the Global Health Observatory. Mortality from cervical cancer (i.e. transition probabilities from “Cancer” state to “Dead” state) was determined by a Weibull survival model that was fit to reported 5-year survival rates for cervical cancer patients from African cancer registries [8]. Digitized Kaplan-Meier curves were used to reconstruct an individual patient dataset, which then was used to fit the survival model using a previously described algorithm [9].

The simulation model was implemented in the C++ programming language and compiled using XCode command-line tools (version 2397). The Rcpp package was used to run simulations from R (version 4.3.2), in which the results were analyzed.

**Model calibration**

The Nelder-Mead optimization algorithm [10] was used to calibrate the disease model parameters to three outcomes: age-specific hrHPV prevalence, crude cervical cancer incidence rate and proportion of cervical cancer caused by HPV types 16/18. Informed by relevant literature, the calibration targets were set for each of the outcome. Log-likelihood was used the goodness-of-fit measure for optimization (with maximization of log-likelihood as the objective). The calibration parameters are described in Table S2. The best-fitting transition probabilities are provided in Table S3.

First, the transition probabilities from hrHPV-negative to hrHPV-positive state for 5-year age groups (11 probabilities in total) were calibrated to reported prevalence rates for 4 age groups. Next, the transition probabilities between hrHPV-negative and CIN states (including progression and regression) were calibrated to a reported crude incidence rate for Uganda. Finally, the model parameter representing the increased risk of progression to pre-cancer for HPV 16/18 types was also calibrated to a target proportion of cervical cancer cases caused by HPV 16/18.

For the calibration procedure, a sample of 100 values for each parameter were used. For the first two steps, which focus on probabilities, beta distribution was used. For the third step, the “increased progression risk” modifier was treated as a normally distributed variable and 20% of the mean was set as SD.

**Table S2. Calibration parameters**

| **Target outcome(s)** | **Target value(s)** | **Target value source(s)** | **Model parameter(s) calibrated** | **Sampling parameters for calibration** |
| --- | --- | --- | --- | --- |
| hrHPV prevalence | Age 25-34: 0.31  Age 35-44: 0.25  Age 45-54: 0.22  Age 55-64: 0.31 | [36] | Age-specific transition probabilities from hrHPV-negative to hrHPV-positive (11 in total) | Beta distribution with initial transition probabilities used as means, 20% of the initial value used as SDs |
| Cervical cancer crude incidence rate | 30 per 100 000 woman-years at risk | [11] | Transition probabilities between hrHPV-positive, CIN1, CIN2, CIN3, and Cancer states (13 in total) | Beta distribution with initial transition probabilities used as means, 20% of the initial value used as SDs |
| Proportion of cervical cancer cases caused by HPV 16/18 | 73.5% | [12] | “Increased risk” modifier for transition probabilities to CIN1/2/3 for HPV16/18 vs other hrHPV | Beta distribution with initial transition probabilities used as means, 20% of the initial value used as SDs |

**Table S3. Calibrated transition probabilities (best-fitting set)**

| ***to***  ***from*** | **hrHPV-negative** | **hrHPV-positive** | **CIN1** | **CIN2** | **CIN3** | **Cancer** |
| --- | --- | --- | --- | --- | --- | --- |
| **hrHPV-negative** | - | 15-19: 0.1742073  20:24: 0.4375693  25-29: 0.3731307  30-34: 0.2620121  35-39: 0.2912969  40-44: 0.2010394  45-49: 0.2078710  50-54: 0.1760468  55-59: 0.1699475  60-64: 0.2796978  65-69: 0.2882833 | 0 | 0 | 0 | 0 |
| **hrHPV-positive** | 0.5034 | - | 0.0760124 | 0.0033408 | 0.0059556 | 0 |
| **CIN1** | 0.4 | 0.1916024 | - | 0.0273075 | 0.0046732 | 0 |
| **CIN2** | 0 | 0.0062401 | 0.0303385 | - | 0.0634340 | 0 |
| **CIN3** | 0 | 0.0025375 | 0.0058699 | 0.0627905 | - | 0.0457616 |
| **Cancer** | 0 | 0 | 0 | 0 | 0 | - |

**Sensitivity analysis parameters**

The parameter values used in the base case analysis are presented in Table 2 of the manuscript. Beta distribution was used with mean as the base case value and the SD was constructed assuming 95% confidence intervals reported in the sources were calculated using Wald method. For these means and SDs, the shape parameters of beta distribution ($\alpha$ and $\beta$) were determined as follows:

$$\alpha=(\frac{1-\mu}{\sigma^{2}}-\frac{1}{\mu})\times\mu^{2}$$

$$\beta=\alpha\times(\frac{1}{\mu}-1)$$

**References**

1. Bulkmans NWJ, Berkhof J, Bulk S, Bleeker MCG, van Kemenade FJ, Rozendaal L, et al. High-risk HPV type-specific clearance rates in cervical screening. Br J Cancer. 2007;96: 1419–1424. doi:10.1038/sj.bjc.6603653

2. Lazare C, Xiao S, Meng Y, Wang C, Li W, Wang Y, et al. Evaluation of Cervical Intraepithelial Neoplasia Occurrence Following the Recorded Onset of Persistent High-Risk Human Papillomavirus Infection: A Retrospective Study on Infection Duration. Front Oncol. 2019;9: 976. doi:10.3389/fonc.2019.00976

3. Dick S, Kremer WW, Strooper LMAD, Lissenberg-Witte BI, Steenbergen RDM, Meijer CJLM, et al. Long-term CIN3+ risk of HPV positive women after triage with FAM19A4/miR124-2 methylation analysis. Gynecol Oncol. 2019;154: 368–373. doi:10.1016/j.ygyno.2019.06.002

4. Denise Zielinski G, Snijders PJF, Rozendaal L, Voorhorst FJ, Runsink AP, de Schipper FA, et al. High-risk HPV testing in women with borderline and mild dyskaryosis: long-term follow-up data and clinical relevance. J Pathol. 2001;195: 300–306. doi:10.1002/path.981

5. Nobbenhuis MA, Walboomers JM, Helmerhorst TJ, Rozendaal L, Remmink AJ, Risse EK, et al. Relation of human papillomavirus status to cervical lesions and consequences for cervical-cancer screening: a prospective study. Lancet Lond Engl. 1999;354: 20–25. doi:10.1016/S0140-6736(98)12490-X

6. Motamedi M, Böhmer G, Neumann HH, von Wasielewski R. CIN III lesions and regression: retrospective analysis of 635 cases. BMC Infect Dis. 2015;15: 541. doi:10.1186/s12879-015-1277-1

7. Loopik DL, IntHout J, Ebisch RMF, Melchers WJG, Massuger LFAG, Siebers AG, et al. The risk of cervical cancer after cervical intraepithelial neoplasia grade 3: A population-based cohort study with 80,442 women. Gynecol Oncol. 2020;157: 195–201. doi:10.1016/j.ygyno.2020.01.023

8. Sengayi-Muchengeti M, Joko-Fru WY, Miranda-Filho A, Egue M, Akele-Akpo M-T, N’da G, et al. Cervical cancer survival in sub-Saharan Africa by age, stage at diagnosis and Human Development Index: A population-based registry study. Int J Cancer. 2020;147: 3037–3048. doi:10.1002/ijc.33120

9. Guyot P, Ades A, Ouwens MJ, Welton NJ. Enhanced secondary analysis of survival data: reconstructing the data from published Kaplan-Meier survival curves. BMC Med Res Methodol. 2012;12: 9. doi:10.1186/1471-2288-12-9

10. Nelder JA, Mead R. A Simplex Method for Function Minimization. Comput J. 1965;7: 308–313. doi:10.1093/comjnl/7.4.308

11. International Agency for Research on Cancer. Uganda Fact sheet. 2021 [cited 7 Apr 2023]. Available: https://gco.iarc.fr/today/data/factsheets/populations/800-uganda-fact-sheets.pdf

12. Banura C, Mirembe FM, Katahoire AR, Namujju PB, Mbonye AK, Wabwire FM. Epidemiology of HPV genotypes in Uganda and the role of the current preventive vaccines: A systematic review. Infect Agent Cancer. 2011;6: 11. doi:10.1186/1750-9378-6-11
